# National Security Law Education in Hong Kong: Qualitative Evaluation Based on the Perspective of the Students

**DOI:** 10.3390/ijerph20010553

**Published:** 2022-12-29

**Authors:** Daniel T. L. Shek, Xiaoqin Zhu, Diya Dou, Xiang Li

**Affiliations:** Department of Applied Social Sciences, The Hong Kong Polytechnic University, Hong Kong, China

**Keywords:** Hong Kong National Security Law, law abidance leadership, course evaluation, focus group evaluation

## Abstract

In this paper, we adopted a qualitative evaluation approach to understand the subjective views of the students on a program on law abidance leadership education covering the Hong Kong National Security Law. The program involves a 3 h lecture and 7 h of self-study on topics surrounding national security. To evaluate the program, we adopted a general qualitative research design to collect data via focus groups using a semi-structured interview guide. Researchers with a doctoral degree and qualitative research experiences conducted six focus groups involving 52 randomly selected students, with 6 to 12 participants per group. Results showed high inter-rater reliability in the thematic analyses. With reference to the questions in the semi-structured interviews, several observations could be highlighted. First, students had different views on different aspects of the lecture, including content, arrangement, lecture notes and assessment. Second, students generally had positive views of teachers’ interaction with students and their teaching performance. Third, students had positive learning experiences and they perceived benefits and value of the program. Fourth, despite the positive comments of the students, some students proposed suggestions for improvement. Finally, the program was not seen as brainwashing by the students. Together with evaluation findings based on four other evaluation studies, the triangulated findings suggest that this program promoted the knowledge about law abidance leadership in the students and nurtured their positive attitudes towards law abidance.

## 1. Introduction

The aim of this study was to examine students’ views on a law abidance leadership program using data collected via the focus group methodology. In opposition to the Anti-Extradition Law Amendment Bill proposed by the Government, serious protests took place in Hong Kong from early summer 2019 to early 2020 [1], which led to serious negative public health consequences [2]. To restore social stability in Hong Kong and to promote national security, the Standing Committee of the National People’s Congress passed the “Hong Kong National Security Law (NSL)”. In Article 10 of NSL, it is stated that education institutions including universities have the responsibility to promote NSL education.

The NSL has much relevance to the quality of life of the public. First, the rule of law has been regarded as an important indicator of quality of life in a place. As argued by Dingake [3], “whether embodied in constitutions, statutes, regulations, executive orders, administrative agency decisions, or court decisions, the law plays a profound role in shaping life circumstances and, in turn, health” (p. 295). Second, with reference to the eight dimensions of well-being proposed by Stoewen [4], law abidance education would promote intellectual well-being (expanding knowledge and skills), emotional well-being (management of emotions surrounding the NSL), social well-being (contribution to the society by following the law), and environmental well-being (understanding how the social environment affects oneself). Third, as the NSL is new to Hong Kong, inadequate and biased understanding would compromise the well-being of Hong Kong people. Unfortunately, studies on quality of life have been dominated by studies on the physical quality of life [5].

There are several difficulties in implementing NSL education. First, it is a politically sensitive and controversial issue that would arouse negative sentiments. Second, NSL education may remind people of political propaganda. Third, as NSL education is brand new to Hong Kong, feelings of uncertainty and anxiety would be created.

To promote university students’ understanding of NSL, we have incorporated NSL education within the leadership development framework in a subject entitled “Tomorrow’s Leaders”. In this subject, we have developed a 3 h module on law-abiding leadership, which covers law-abiding leadership, concepts of national security and the Hong Kong National Security Law [6]. In addition to the 3 h of lecture, students are also required to have 7 h of study on their own. In addition to the lecture material, we also distribute 60 lecture notes to the students. To assess the students’ understanding of the subject matter, they are required to take an assessment with 20 multiple-choice items with a passing standard of 16 correct answers. Multiple attempts are allowed.

We implemented NSL education in Semester 1 and Semester 2 of the 2021/22 academic year. Each semester, we conducted a post-lecture evaluation after the lecture. For Semester 1, survey results (N = 890) showed that students agreed that the lecture was able to increase their understanding of the concept of national security and the Hong Kong National Security Law, and they were ready to serve as socially responsible leaders [6]. Again, we conducted a post-lecture evaluation after the lecture in Semester 2 (N = 914) which generated similar findings [7]. In addition to understanding the views of students, we also examined the views of six teachers teaching NSL education after Semester 1 via a qualitative study. In line with the studies based on students, this study suggests that the program promoted law abidance knowledge and attitudes in students [8]. Similarly, we collected qualitative data from all teachers teaching NSL education after Semester 2 of the 2021/22 academic year. The findings triangulated those findings based on students and teachers [9]. In short, four published papers have documented the positive evaluation findings of this program.

Although existing evaluation findings based on the feedback of students and teachers suggest that NSL education promotes student well-being in terms of their understanding, attitudes, and behavioral intentions surrounding the NSL, there are several limitations. First, as student surveys were conducted after the lecture only, students’ experiences about the 7 h self-study and assessment were not covered. Hence, it would be more complete if we could obtain some feedback after the completion of the entire course. Second, we did not randomly select the students in the post-lecture evaluation. Students participated in the two studies in a voluntary manner only. Third, although we collected qualitative feedback from the students in the surveys, we could not ask follow-up questions about their comments. Fourth, as there is a belief among some people that NSL education is a “brainwashing” tool (which would be bad for the political well-being of the students), there is a need to understand whether the related education is regarded as “brainwashing” from the perspective of the students.

With the above background, we conducted a qualitative study using the focus group method after Semester 1 of the 2021/22 academic year. In the study, we attempted to understand the learning experience and perceptions of the students on NSL education embedded in law-abiding leadership education (3 h lecture plus 7 h self-study). Specifically, we explored the following research questions:

1. What are the views of the students on the lecture, including its content, arrangement, atmosphere, interaction and the teacher?
2. What are the students’ views on the 60 lecture notes?
3. What are the students’ views on the assessment?
4. What are the experiences of the students on the lecture, including their views on the benefits and understanding of the subject matter?
5. What are the students’ perceptions of the views of classmates on National Security Law education?
6. Do students regard National Security Law education as “brainwashing”?

Consistent with the common organization of a scientific research article, after outlining the method of the study, we present the findings with reference to the research questions. Then we discuss the contributions and limitations of the study.

## 2. Materials and Methods

To understand the views of the students, we adopted a qualitative research strategy in this study. According to Tomaszewski, Zarestky and Gonzalez [10], there are different types of qualitative research, including ethnography, narrative, phenomenology, and case study. In this study, we adopted a case study approach focusing on the perceptions of the students on the law abidance leadership program. Renjith et al. [11] argued that “case studies are best suited for the understanding of case(s), thus reducing the unit of analysis into studying an event, a program, an activity or an illness” (p. 4), with focus group discussion as a common data collection method. According to McLafferty [12], focus group interview is a data collection strategy.

As pointed out by Tümen-Akyıldız and Ahmed [13], “compared with other collecting data techniques such as questionnaires, observations, and so on, group discussions can be a valuable source to explore the internal views and emotions of individuals, interviewing with participants is highly suitable for knowing because it focused on feelings, thoughts, perceptions, sensitive matters, experiences, and knowledge of the members” (p. 7). However, because of its fluid nature, researchers have criticized the focus group method. For example, Nyumba et al. [14] pointed out that there are methodological gaps in the existing studies, including failure to report justifications for using the methodology, number of groups and sample size in different groups. Based on a review of the literature, Vermeire et al. [15] also pointed out the inadequacies in focus group studies and proposed a checklist to ensure the quality of focus group discussion.

In a review of qualitative evaluation studies, Shek et al. [16] proposed 12 principles to increase rigor in qualitative evaluation studies. These include a clear articulation of the philosophical orientation of the study, justification for the sample recruited and the sample size, description of how data are collected, consciousness of biases and preoccupations involved, and ways to minimize biases, measure of reliability, triangulation, peer checking and member checking, the existence of audit trails, negative evidence, and recognition of limitations of the study. We adopted these principles as far as possible in this study. First, we adopted a general qualitative orientation focusing on the importance of narratives and themes, close contact between the researcher and the participants, and non-numerical analyses of the data. Second, we consciously used a large sample to maximize the variation in the views of the students. Moreover, we randomly selected participants to increase the representativeness of the findings. Third, we also acknowledged the possible biases involved. As such, we proposed several measures to minimize such biases. First, four researchers and several research assistants participated in the study to minimize related biases. Second, three colleagues were involved in data analyses and we conducted inter-rater reliability on the positivity of the derived themes. These measures are further discussed in the Discussion section.

### 2.1. Participants and Procedure

To promote university students’ understanding of NSL, we have incorporated NSL education within the leadership development framework at the authors’ university in Hong Kong. The university was founded in 1937 and it has become the largest government-funded university in Hong Kong in terms of the number of students. As of 2021, the university has 27,214 students, including 984 sub-degree students, 15,155 undergraduate students, and 10,431 postgraduate students. Among these students, 19,452 (71.48%), 6676 (24.53%), and 1086 (3.99%) come from Hong Kong, other places in China (mainland China, Macau, and Taiwan), and overseas countries and regions, respectively.

In the first semester of the 2021/22 academic year, the leadership subject with law abidance leadership education was offered to 18 classes of Year 1 students. After the completion of the subject, subject teachers randomly selected three students in each class using random numbers. Specifically, the subject teacher used the random number function in Microsoft Excel to generate random numbers between 0 and 1 for students in the class and select the three students with the three largest numbers. Then, subject teachers invited the selected students to participate in focus groups to be conducted in December 2021. If the invited student did not agree or did not have time to join the focus group, the subject teacher invited an alternate student selected randomly from the remaining students using the identical method. A total of 71 students in 18 classes were invited, and 52 students participated in the focus group (two students dropped before the focus groups because of a time clash caused by personal issues), resulting in a response rate of 73.2%.

All participants provided their written consent to join the focus groups. Six focus group interviews were conducted in December 2021. While three focus groups were conducted in Cantonese (N = 26), two focus groups were conducted in Mandarin (N = 17), and one group was conducted in English (N = 9). The number of students in each focus group ranged between six and eleven, which is consistent with the recommended group size (6 to 12 participants in the public health field [17]). Because of social distancing measures during the pandemic, each interview was conducted by one assistant professor with a Ph.D. degree via ZOOM. One research staff was also present at the interview to take care of the logistics and arrangement of the focus group interview. All interviews were recorded upon participants’ consent. In the focus group discussion session, we used a semi-structured interview guide to understand the subjective experience regarding the law abidance leadership program. The duration of the interviews ranged between 60 and 180 min. All student participants received a HK$100 voucher per hour as a token of appreciation for their time and efforts after the completion of the interviews. We obtained institutional ethics approval before data collection. As the data collection was carried out after the completion of the subject, the participants were assured that their grades would not be affected by their responses. In addition, the interviewers did not teach the participants and did not know their identities.

Regarding the conceptual framework for the evaluation study, as studies showed that evaluations of education programs are commonly centered around perceptions of the program, instructors, benefits, and experience [18,19], we asked several questions based on this conceptual scheme. First, we asked students’ views on the different aspects of the lecture (content and interaction, etc.), notes and assessment. Second, we explored students’ perceptions of the teacher. Third, we examined the students’ views of the benefits and necessity of the program. Finally, to understand the experiences of the students, we also examined their experiences, perception of the related experiences of their classmates, and whether the program was “brainwashing”. Table 1 presents the questions in the semi-structured interview guide.

To encourage students’ participation by minimizing the sensitivity in participation and collecting as less personal information as possible, we did not collect other demographic information other than age and gender. The results did not reveal any connections between students’ opinions and their age or gender.

### 2.2. Data Analysis

We focused on the verbal data of the group discussion (i.e., narratives), which is a common practice in focus group studies. We did not conduct any video analysis because the interviews were conducted via ZOOM. For the data analysis framework, we used thematic analyses to analyze the narratives guided by the different interview questions [20]. According to Braun and Clarke [21], “thematic analysis is a method for identifying, analyzing and reporting patterns (themes) within data. It minimally organizes and describes your data set in (rich) detail” (p. 79) and there are six steps involved. These include: familiarization with the data; generation of initial codes; identification of themes; review of themes; ongoing refinement and clear definition of themes; and publication of reports. According to Vaismoradi et al. [22], “thematic analysis involves the search for and identification of common threads that extend across an entire interview or set of interviews” (p. 400).

In the present study, after fully transcribing each focus group interview, we identified patterns under each interview question. For most interview questions (e.g., lecture content, teacher, benefits, lecture notes and assessment, views on law abidance leadership education), we organized the patterns primarily in terms of whether the themes were positive or negative themes (i.e., the positivity of patterns). For example, regarding the benefits of law abidance leadership education, students’ comments (e.g., “useful”, “helpful”, “insightful”, “valuable”, “beneficial”, and “promoted my critical thinking”) could be regarded as “positive” narratives under the theme of “positive benefits”. After the research colleagues determined the initial themes, the researchers finally checked the themes derived.

To understand the reliability of the analyses, three research assistants re-coded 20 randomly selected narratives to determine the inter-rater reliability of the narratives, particularly their positivity [16]. Results showed that three coders agreed on 18 comments (inter-rater reliability = 18/20 = 0.9), and two of the three coders agreed on two other comments. The findings demonstrated a very high level of inter-rater reliability in the analyses. For illustration purposes, we present two examples here. First, regarding the narrative that “my lecturer is very enthusiastic and very good. (He/she) will promote the classroom atmosphere and ask more approachable questions”, three raters rated the narrative as a positive theme. Second, regarding whether NSL education is brainwashing, three raters rated the following narrative to be positive: “I don’t think this is brainwashing because this is only one lecture introducing Chinese history and the NSL. There are the basic things that we need to know when studying in Hong Kong”.

## 3. Results

Among the 52 participating students, 25 (48.1%) were female students and 27 (51.9%) were male students. Their ages ranged from 18 to 31 years old, with an average age of 19.5 years old. In terms of the sample size, it was above the mean value of the size of focus groups in 220 studies reviewed by Carlsen and Glenton [23]. In addition, we randomly selected the students, which helped enhance the generalizability of the findings [24]. As some themes were not related to the positivity of the comments (e.g., the class should be arranged earlier—timing of the lecture), they were classified as “neutral comments” and examples were given.

With reference to the questions in the interview guide, we present the findings in the following sections. Examples of the related positive narratives are presented in the respective tables.

### 3.1. Perceptions of the Lecture on Law-Abiding Leadership

First, we observed some positive themes in the lecture content, including “straight-forward” and “promotion of understanding”. However, some students expressed that “the focus of the lecture was not clear”, “coverage of Chinese History was too much”, and “linkage between History and national security was not clear” (i.e., negative themes). Some neutral themes included “this is something new to me” and “surprise to include this topic in a leadership course”.

Second, regarding arrangements such as the medium of instruction, while there are some positive themes (e.g., “very good”, “good arrangement”, “perfect lecture”, and “appropriate”), some negative themes are also identified (e.g., “easier to study Chinese History in Chinese”, “increase the number of classes”, and “time too short”). For the duration of the lecture, while some students commented that the lecture on NSL might be “too short” and “not all the learning content was clearly explained”, a student commented that “the timing of the lecture was good”. An additional neutral theme is “not understanding the linkage between Chinese history and NSL”.

Third, there are mixed findings on the class atmosphere. While some students shared that the lecture atmosphere was “relaxing”, “interesting”, “not stressful”, “comfortable”, and “students are willing to interact” (i.e., positive themes), some students reflected that the atmosphere was “bored”, “quiet”, “information load” and “long lecture duration” (i.e., negative themes). Nevertheless, no participant mentioned that the program was anxiety- and stress-provoking. Neutral themes include “there were fewer students in class” and “challenging to discuss online”.

The fourth observation concerns the interaction in class. For teacher-student interaction, students had overwhelmingly positive experiences: “teachers invited students to answer questions”; “teacher shared their opinion on the topics”; “connect students in the classroom and online” (i.e., positive themes). For interactions amongst the students, some students shared that they “had more discussions with their classmates than in other lessons” and “had some interactions amongst the students” (i.e., positive themes). On the other hand, many students commented that there were “embarrassment” and “limited discussion” (i.e., negative themes). Based on the narratives, four factors were identified as contributing to the negative themes, including the sensitivity of the topic (e.g., fear of being video-taped), hybrid teaching, unfamiliarity with the topic, and language barrier. Finally, some students mentioned that interaction in their groups was high while interaction in other groups was low, hence suggesting that interaction depends on group nature (i.e., neutral theme). Additional neutral themes included “teachers’ attitude shaped class discussion” and “students may have difficulty in answer questions in English”.

Regarding the performance of teachers, the narratives were overwhelmingly positive. In terms of preparation, most students agreed that their teachers were “well-prepared”, “presented the teaching materials clearly”, “made clarifications on students’ questions”, and “gave guidance when needed”. For teaching strategies, students found that their teachers “used different strategies to motivate students”, such as “used questions to guide students’ thinking and reflection”, and they also “illustrated with daily examples or personal experience to explain the concepts”. Students also mentioned that their teachers were “caring” and “respect students”, such as “actively engaged in discussion”, “paid attention to students’ responses”, “provided feedback”, and “respected students with different views on the topic”.

Some narratives associated with students’ perceptions of the lecture and teachers can be seen in Table 2. Although there were mixed views on different aspects of the lecture, students generally had positive comments on teacher-student interaction and teacher performance.

### 3.2. Lecture Notes and Assessment

Table 3 shows some narratives on students’ views on the lecture notes and assessment. Students generally agreed the notes were “useful in building knowledge on NSL and Chinese History”, “logical”, “systematic” and “interesting”. They also found them “helpful in passing the quiz” (i.e., positive themes). On the other hand, some students found the notes to be “too long to read” and “difficult to understand Chinese History in English” (i.e., negative themes). Surprisingly, a few students expressed that they “did not know the existence of the notes”, although this is clearly included in the course outline, and teachers repeatedly mentioned this in the first seven lectures.

Students had different opinions on the quiz. Some students regarded the quiz as “difficult”, “too detailed”, and “tricky” (i.e., negative themes). On the other hand, some students found the quiz to be “helpful”, “appropriate”, and “consolidated students’ knowledge” (i.e., positive themes). Finally, there are different views on the passing mark. While some views suggest the need to “lower the passing requirement”, some students suggested “using a higher passing mark”. Some students remarked that “the test was not difficult”.

### 3.3. Perceived Lecture Experience and Benefits

Students had overwhelmingly positive experiences with the lecture, including “great experience”, “useful”, “good opportunity to understand NSL”, “fulfilled”, and “interesting” (i.e., positive themes). Moreover, many participants perceived the program as “beneficial” in three areas. The first area is in terms of understanding law abidance leadership, such as “giving students basic knowledge on NSL”, “enhancement of students’ understanding of NSL”, and “promoting students’ learning motivation”. The second area is the promotion of positive attitudes such as “learned how to respect others”, “promoted one’s national identity”, and “determination not to break the law”. Finally, students remarked that the program helped “nurture their critical thinking” and “sharpen their mind”. Table 4 shows some narratives about the positive lecture experience and perceived benefits of the program. Neutral themes included “the experience would be better if conducted via face-to-face mode” and “coverage of Chinese history was factual”.

### 3.4. Views on Law-Abiding Leadership Education

Students generally agreed that university students should understand NSL because it is “necessary” and “practical”. Students also agreed that understanding NSL is “students’ responsibility” that can “help them integrate into Hong Kong culture”, “understand different views involved”, and “promotion of interaction amongst students” (i.e., positive themes). However, some students who had reservations mentioned that “it is not practical” and there is “no need to learn so much Chinese History” (i.e., negative themes). Furthermore, students felt their classmates had positive views on the subject matter. For local and international students, they said that some of their classmates agreed that students should have a basic understanding of National Security Law. In particular, students from mainland China strongly agreed that it was very necessary to have NSL education. However, a student commented that his/her classmates “did not want to do the MCQs” (i.e., negative theme). Finally, some respondents were “not sure about the views of the classmates”, “it depended on groups” and “I did not talk to my groupmates” (neutral themes). Table 5 shows some examples of the related narratives.

### 3.5. Area(s) for Improvement/Suggestions

Although students generally had positive experiences and views on the law abidance leadership program, several areas of suggestions for improvement emerged from the qualitative data. For the lecture content, some students suggested “increasing the proportion on National Security Law”, “focusing on NSL”, and “adding more information on Hong Kong History”. For the arrangement, some students suggested “using Chinese as the medium of instruction” or “providing the lecture in different languages”. Some students suggested “having more lectures for a deeper understanding” and “separated NSL and Chinese History into two lectures”. As far as instruction is concerned, students suggested that “teacher could use more examples and multi-media materials” and “teachers should pay more attention to the different pace of students in the face-to-face and online settings”. In addition, some students suggested having “more interactive activities”, “using applications like Kahoot”, “grouping students”, “designing some team building activities at the beginning of the lecture”, and “dealing with students’ concerns on revealing their identity”.

### 3.6. Is Law-Abiding Leadership Brainwashing?

The participants generally disagreed that the lecture was brainwashing because the lecture content (history and National Security Law) was “basically factual in nature” and “students had independent thinking skills”. Two students mentioned that whether it was brainwashing depends on the “definition of brainwashing”. Table 6 shows some narratives surrounding this topic.

## 4. Discussion

Regarding the evaluation of the law abidance leadership education program, we employed a focus group methodology to understand the students’ perceptions and experiences. While there were different views on different aspects of the lecture (including content, arrangement, lecture notes, and assessment), positive views on teacher performance, learning experience, perceived benefits, and need for law abidance leadership education were observed. Most importantly, the findings clearly suggest that students did not feel that the related education was brainwashing. This finding is important because there are criticisms about the implementation of the National Security Law in Hong Kong. Shek, Dou, Zhu and Li [7] pointed out that there are polarized views on the implementation of the Hong Kong National Security Law. While there are views suggesting that the NSL is legitimate and many countries have laws on national security, Clift [25] pointed out that NSL would undermine civil liberty and political rights in Hong Kong. Hence, the present study provides some pioneer qualitative findings on this issue.

The students made some suggestions for improvement. While we welcome such suggestions, there are several difficulties in implementing their suggestions. First, there are different views among students on the recommendations. Second, there are pedagogical considerations for some issues, such as whether there is a need to focus on modern Chinese history. Our standpoint is that coverage of modern Chinese history would help students appreciate the importance of national security and the need for Hong Kong National Security Law, and covering NSL without the historical context is a deficient approach. Third, there is the issue of practicality. For example, as the University adopts English as the medium of instruction, the suggestion of using Chinese is not practical. Finally, some students made suggestions because they were unfamiliar with the subject content and requirements.

There are several issues one should consider when understanding the present findings. The first issue is the use of the focus group method in evaluation. From a post-positivistic perspective, one may argue that focus group studies are subjective and “non-scientific” because we only focus on the views of the students. Nevertheless, it is noteworthy that focus group studies have been strongly emphasized as a good strategy to understand the views of different stakeholders as the findings can generate rich and fluid information about the problem under focus [24,26]. In reality, the focus group method has been widely used in public health [17,27,28] and higher education [29]. In particular, evaluators have commonly used focus group discussions to understand program effects. Brandl et al. [30] remarked, “including open-ended focus groups can provide rich solution-based feedback that makes this a worthwhile tool to add to the evaluation toolbox” (p. 5). As pointed out by Ansay, Perkins and Nelson [31], “although focus groups continue to gain popularity in marketing and social science research, their use in program evaluation has been limited” (p. 310) and “the use of focus groups in evaluation research is relevant to the goals of policymakers and program administrators whose goal is to better serve their constituents and stakeholders” (p. 315). Sim and Snell also pointed out that focus group evaluation was neglected in physiotherapy, which could help understand the perceptions of patients [32].

The second issue involves criticisms of the rigor of focus group interviews. To increase the rigor of this study, we consciously upheld the 12 principles outlined by Shek, Tang and Han [16] as far as possible. In the Introduction section, we clearly stated that this is a focus group study adopting a general qualitative approach, and we justified the sample size and how the sample was recruited. In this paper, we also clearly described how the data were collected. As we were conscious of the possible biases, we had several measures to deal with this issue. First, a team of researchers and research-supporting staff were involved in different stages to minimize systematic biases. Second, several colleagues were involved in the data analysis. Third, we conducted inter-rater reliability in this study. To understand the findings from different perspectives, we triangulated the findings concerning four other evaluation studies (see below). We also presented and examined negative evidence and systematically kept the data, which permitted audit trails. Finally, we acknowledged the limitations of this study.

The third issue is whether the findings align with the previous studies. In addition to this focus group study, we have conducted four other evaluation studies for the program in the 2021–2022 academic year. First, we conducted a post-lecture evaluation for the Semester 1 program. Results showed that the respondents were positive about the program, teacher, and benefits. Students also agreed that they would try their best to be law-abiding responsible leaders [6]. Second, we conducted a post-lecture evaluation for the Semester 2 class as well. Consistent with the first study, students appreciated the benefits of NSL education and showed their readiness to be law-abiding leaders [7]. Third, we examined the views of the teachers teaching the program in Semester 1 of the 2021–2022 academic year. Basically, teachers regarded the program as meaningful and beneficial to students [8]. Finally, teachers teaching in Semester 2 of the 2021–2022 academic year were also invited to reflect on their teaching experiences. Again, the findings are consistent with the first three studies. The teachers saw that the program benefited the students, and the student learning experience was positive [9].

From a triangulation perspective, we used different research methods (questionnaire, self-reflection, and focus groups), different data (quantitative and qualitative data), and different informants (students and teachers) in these studies. The overall picture derived is that students had positive learning experiences, and they had positive perceptions of the benefits (such as promotion of their knowledge on law abidance leadership) and value of the program. Caillaud and Flick [33] highlighted the value of doing focus groups to triangulate findings within a study or across studies. In the present context, the present focus group study triangulates the findings obtained in four previous studies. Certainly, it would be helpful to combine the different datasets across these studies. However, in view of the sensitive nature of the topic, we did not collect personal identifiers of the participants. As a result, we cannot match the participants across these studies.

The fourth issue is that, in conjunction with other evaluation studies mentioned above, the present findings suggest that the program promoted the well-being of the students. As the topic is politically sensitive, the common myth is that it may create negative emotions for the students. However, the present findings showed that students did not find the program anxious and stressful. Instead, they felt relaxed and comfortable. Moreover, the positive learning experience and perceived benefits contributed to the academic well-being of the students, such as enhanced motivation to learn and appreciation of the learning involved. The findings showed that students viewed that the program promoted their NSL knowledge and cultivated positive attitudes (e.g., “care about others” and “enhanced love for the Nation”) and skills (e.g., “critical thinking skills” and “sharpen one’s mind”). Obviously, if students do not understand NSL and they do not follow NSL, they will engage in anti-social behavior which is an indicator of social ill-being. In addition, they have to face undesirable consequences (e.g., going through the prosecution process), which would impair their well-being. Regarding different dimensions of health [34], the present study showed that law abidance leadership education promoted cognitive health (increase in NSL knowledge), social health (well-being related to social phenomena), and environmental health (well-being related to political events) of the students. Based on the concept of social well-being of Keyes [35], NSL education also contributes to social integrity (e.g., safe community) and social contribution (e.g., abiding by the NSL). Conceptually, there are views suggesting that rule of law is a foundation of wellness [36].

Fifth, although the present study did not aim at generating a theory on law abidance leadership education, the present findings have several implications for future research. First, the present findings suggest that there is a need to further understand the ambivalence of students in expressing their views during class. As NSL is new to Hong Kong, there is a need to explore this issue further. Second, how lecture content, arrangement and teacher-student relationship may shape the learning experience of the students deserves further investigation. Third, although there is no sign showing that law abidance leadership program creates negative impacts on student well-being, we should further explore how teaching politically sensitive topics may have impacts on students. Finally, as there are few studies on education on politically sensitive topics, the present study contributes to the related knowledge base. Lowe and Jones [37] pointed out the difficulties in teaching controversial topics. Studies also pointed out the tension involved in teaching politically sensitive topics in school settings due to teachers’ negative views and the complexities of issues involved [38,39]. Nevertheless, as commented by Heath et al. [40], “addressing sensitive content is a professional responsibility for teachers, disciplines, and universities” (p. 5). To uphold such a responsibility, it is important to understand the views of different stakeholders, particularly those of students on education on politically sensitive topics.

Although the findings of the study are generally positive, we should note several limitations intrinsic to the study. First, we conducted the study at the end of Semester 1 only. It would be helpful to conduct more related studies in the future. Second, we conducted six focus groups only. Although the students were randomly selected, it would be more illuminating if more students were recruited. Third, as group context may constrain the participants’ views, it would be more holistic if we could conduct additional individual interviews in the future as well. Finally, to understand the program further, there is a need to conduct process evaluations [41] to understand the process of delivering the NSL content and the quality of the classes. If possible, it would be helpful to combine quantitative and qualitative data across different studies. 

## 5. Conclusions

Despite the above limitations, the present study contributes to our understanding of the views of the students on the law abidance leadership program in response to the Hong Kong NSL. In particular, it is noteworthy that different measures were utilized in this focus group evaluation study to promote its methodological rigor, which is rare in focus group studies. In conjunction with previous findings [6,7,8,9], the present findings suggest that students perceived their learning experiences in the law abidance leadership education program and the benefits of the program in a positive way. The findings also suggest that the program promoted the students’ well-being.

## Figures and Tables

**Table 1:** Interview guide for the focus group study.

No	Question
1	What are your views on the following aspects of the lecture? Lecture content Classroom atmosphere Interaction between the teacher and students Interaction amongst the students Teacher performance Benefits of the lecture Areas to be improved (if any)
2	What are your views on the 60 lecture notes? What have you learned from the material? Do you enjoy reading the materials?
3	What are your views on the assessment involving 20 multiple-choice questions? Is it too easy or too difficult? Can it consolidate your knowledge about the topics covered in this lecture and the lecture notes?
4	Taken as a whole, what are your experiences about the lecture on Law Abidance Leadership? Please share your experience.
5	Do you think university students should have a clear understanding of the concept of national security, the National Security Law, modern Chinese history and major humiliation historical events?
6	What are the views of your classmates on National Security Law education? Do they think it is reasonable to require students to receive the related education?
7	There is the view saying that National Security Law education is brainwashing. Based on your experience of this lecture, reading of the lecture notes and assessment, do you have the experience of being “brainwashed”?

**Table 2:** Examples of positive narratives of the students.

Lecture Content (14 Positive Comments and 19 Negative/Neutral Comments)
*“I am positive about the lecture, which is conducive to my understanding of the law, can avoid the wrong law, and can also deepen my understanding of the law. I will not be affected by the negative remarks about the law…”* *“I think it’s like kind of good information … it’s like making you have a better understanding like what you should do and what you shouldn’t do.”* *“I think the content is quite OK. That is, everything is clear, and the material is introduced step by step. I do not feel there is anything bad about the lecture.”*
Classroom Atmosphere (10 positive comments and 11 negative/neutral comments)
*“I think this class is relaxed. There are breaks at the appropriate time so that students can relax and take rest. I think this class is relaxing.”* *“I think the atmosphere is good … the atmosphere of the class is quite active which is good.”*
Interaction between the Teacher and Students (9 positive comments)
*“My professor is very proactive, always checking student feedback and whether students understand the concepts. My teacher will ‘catch us’ during the discussion time in every group.”* *“I think the teacher seriously considered the feelings of students taking online and offline classes, and students are encouraged to speak up…. there was a classmate who did not want to speak up, the teacher encouraged the student to speak up.”*
Interaction amongst the Students (8 positive comments and 64 negative/neutral comments)
*“We have interaction with mainland Chinese students, Hong Kong students, and overseas students. We also have online and offline interactions. My feeling about this class is quite good, without the experience of nervousness and heaviness that I originally thought. Overall speaking, it is great.”* *“We are interactive in our discussion. Besides discussion on the content and examples, we also share the issues over which we feel confused.”*
Perceived Performance of the Teacher (40 positive comments)
*“I feel that the teacher’s preparation is very adequate, including much work spent on the lecture content and illustrations.”* *“I think our teachers are particularly well prepared, including the course content and the expression of each point output … the teacher has done a lot of work on this aspect.”* *“I think my teacher is very good all the time like delivering very good content and the specific information on what you should know. So, I think it’s very easy to understand and follow and some activities.”* *“… My lecturer is very enthusiastic and very good. (He/she) will promote the classroom atmosphere and ask more approachable questions.”* *“My lecturer used various examples to help illustrate the point she was making, which I found helped me understand the topics more…it made it a lot easier for me to gauge the various topics that were being discussed... helped us apply it to real-life situations, which I thought was very useful.”* *“The teacher also helped to connect the students. I feel that my teacher cared about every student and my teacher is a very responsible teacher.”* *“When almost at the end of the lecture, the teacher explained why we need this lecture. The argument is that even if you do not agree, students have to learn how to follow the law because this is an important matter. The teacher used a language that would reduce the resistance of those students who do not really like NSL.”*

**Table 3:** Narratives of positive views on lecture notes and assessment.

Lecture Notes (24 Positive Comments and 29 Negative/Neutral Comments)
*“I think the arrangement of the handout is actually very good, because in fact, the purpose of the after-class exercises is not really for the exam, but to let the students have some understanding of these contents …* *“In the process of reading the lecture notes, we have also gained knowledge. When I need to use it to answer some questions, I personally feel ok, although I am not the kind of person who likes reading history, but I read the history as stories, so I feel that it is acceptable.”*
Assessment (5 positive comments and 3 negative/neutral comments)
*“I think it’s still a little bit difficult, as far as I’m concerned. I did it five times to pass it. Some of the questions are too word bound. It feels like it’s not simply a test of your knowledge or content, it also tests your understanding of the word.”* *“Well, I think the Multiple-Choice quiz test is OK. Yet it takes about half an hour to finish it… The lecture notes help a lot. For some questions in the multiple-choice quiz, it is quite hard to find answers. We need to read the lecture notes, carefully and detailed to find the answer. Yes, some are tricky.”* *“The multiple-choice test can motivate students to search for more information after class, get the answers and go back to Blackboard to study the lecture notes, and to consolidate. That is, to protect oneself from breaching the law. Actually, it is very helpful.”* *“I think it is a bit too much to ask students to get correct answers out of 20 multiple choice questions. I have made several attempts before I passed the quiz.”* *“I think the multiple-choice questions are easy because there are many straightforward questions. If we set a low passing limit, students may not have a deep understanding of NSL. Maybe we should set the passing mark to be 18 out of 20.”*

**Table 4:** Narratives of positive views on the experience in the lecture and perceived benefits.

Experience in the Lecture (55 Positive and 6 Negative Comments)
*“I think the whole lecture in Tomorrow’s Leaders is really useful. It gives us a chance to know more about ourselves, and what kind of skills we need to develop in the universities. Yeah, and also learn like the laws in Hong Kong…”* *“Uh, I want to talk about the whole course I really enjoy Tomorrow’s Leaders course. I really enjoy getting the knowledge I don’t know from my teachers. So yeah, I’m super happy and I wish that I will meet this teacher again in the future.”* *“I feel that the lecture and notes are comfortable.”* *“I understand the relationship between leadership and law after taking this lecture.”*
Perceived benefits (40 positive comments)
*“I learned a lot … I usually do not understand the consequences of violating the law. After attending this lecture… it was written well and clearly … it helps a lot.”* *“Well, being a foreigner, I did not hear much about the national security law. So, coming to this lecture definitely gave me a brief idea of what this national security law is about. So, I think that will be helpful for me, teaching me how to stay out of trouble.”* *“I think the biggest thing is to know history because I didn’t study history before… It is good to know the history of different times.”* *“I think it will expand my thinking more, more critical thinking. From the beginning, when I was in my third year of high school, I knew about the National Security Law. I just thought it was really good. Then through this course, I get a deeper understanding of what it really is. Then there is the difference between helping individuals and helping the State … Then I feel like I’m going to think more multi-dimensionally, and that’s what I’m bringing. Thanks, teacher. Good thanks.”*

**Table 5:** Narratives of positive views on law abidance leadership education program.

Views on Law Abidance Leadership (20 Positive Comments and 11 Negative/Neutral Comments)
*“As a local Hong Kong student and a Chinese, we should know Chinese history so that we can develop our national identity and national affiliation, which would motivate me to protect the national security of this country.”* *“Yeah, necessary, so everyone needs to learn, especially since we are studying in Hong Kong, and we must know the laws and the things that happened in Hong Kong because it is related to our life and our safety. Yeah, that’s how.”*
Perceptions of the views of the classmates (20 positive comments and 10 negative/neutral comments)
*“From what I can gauge from my group mates, I think that they see the value in learning this because some of the information that we learned in the lecture, is not really information that you learn every day. It’s kind of more detailed and more thorough, and I think that they see the value in learning that information.”* *“I have talked about this topic with my friends from the Mainland, and they all feel that this is a very necessary arrangement because it can make us more aware of what the content of the national security law is, but I don’t know what the local students think, we mainland students still feel that it is very, very necessary, and we must have this special class to introduce this national security law.”*

**Table 6:** Narratives of positive views on whether law abidance leadership education is brainwashing.

Is Law Abidance Leadership Brain Washing? (42 Positive Comments)
*“I think that in the classroom, including the teacher and the content of the class, there is no promotion of a subjective idea; in fact, it is only some laws and examples of harmful behavior in society, these things do not have a subjective view, but indeed destructive behavior, so I don’t think it is said that the brainwashing point of view is transmitted.”* *“I also think that this is not brainwashing, just because, for example, there are some group discussion sessions in the classroom, there are these opportunities for us to think for ourselves, so we can accept that knowledge through our own thinking, so I don’t think it is brainwashing.”* *“I feel that there is no brainwashing because the lecture time is short, and the concepts are legal facts. Actually, university students have independent thinking. Hence, I do not think I have been brainwashed. Besides Year 1 students, probably Years 2, 3 and 4 students should acquire such knowledge.”*

## Data Availability

The data presented in this study are available on request from the corresponding author. The data are not publicly available due to privacy.
